# Comparison of Postoperative Changes in Inflammatory Marker Levels Between Transthoracic and Transcatheter Device Closures of Atrial Septal Defects in Children

**DOI:** 10.21470/1678-9741-2019-0207

**Published:** 2020

**Authors:** Zhi-Nuan Hong, Jiang-Shan Huang, Kai-Peng Sun, Zeng-Rong Luo, Qiang Chen

**Affiliations:** 1 Department of Cardiovascular Surgery, Union Hospital, Fujian Medical University, Fuzhou, People’s Republic of China.

**Keywords:** Inflammation, Postoperative Period, Heart Septal Defects, Atrial, Cytokines, Procalcitonin, Child

## Abstract

**Objective:**

To explore the postoperative changes in inflammatory markers in children who underwent device closure of an atrial septal defect (ASD) via a transthoracic or transcatheter approach.

**Methods:**

The experimental and clinical data were retrospectively collected and analyzed for a total of 53 pediatric patients between September 2018 and December 2018. According to the different treatments, 19 patients who underwent transthoracic device closure were assigned to group A, and the remaining 34 patients who underwent a transcatheter approach were assigned to group B.

**Results:**

All patients were successfully occluded without any device-related severe complication. Compared with the preoperative levels, the postoperative levels of most inflammatory cytokines in both groups were significantly increased and reached a peak on the first day after the procedure. The level of postoperative inflammatory cytokines was significantly lower in group B than in group A. In addition, there was no significant difference in procalcitonin before and after the transcatheter approach.

**Conclusion:**

Systemic inflammatory reactions occurred after transthoracic or transcatheter device closure of ASDs in pediatric patients. However, these inflammatory reactions were more significant in patients who underwent a transthoracic approach than in patients who underwent a transcatheter approach.

**Table:** 

Abbreviations, acronyms & symbols				
ASD	= Atrial septal defect		POD3	= Third postoperative day
CPB	= Cardiopulmonary bypass		PreOP	= Preoperative day
CRP	= C-reactive protein		SIRS	= Systemic inflammatory response syndrome
IL-6	= Interleukin-6		SPSS	= Statistical Package for the Social Sciences
PCT	= Procalcitonin		TTE	= Transthoracic echocardiography
POD1	= First postoperative day		WBCs	= White blood cells
POD2	= Second postoperative day			

## INTRODUCTION

An atrial septal defect (ASD) is a common congenital heart disease that accounts for 6%-10% of all congenital heart diseases cases^[1]^. Traditional surgical repair has been widely used and efficient for treating ASDs; however, this approach is still associated with a large incision, a slow postoperative recovery, and the need for assistance with cardiopulmonary bypass (CPB)^[2-4]^. In the last 20 years, transcatheter device closure and minimally invasive transthoracic device closure guided by transesophageal echocardiography or transthoracic echocardiography (TTE) have been widely used for secundum ASDs, especially in China^[5,6]^. Many articles have discussed the changes in inflammatory markers after the correction of congenital heart diseases^[7-9]^. However, few studies have focused on the changes in inflammatory markers after the device closure of an ASD. In our center, the inflammatory markers commonly used in clinical practice are C-reactive protein (CRP), white blood cells (WBCs), interleukin-6 (IL-6), and procalcitonin (PCT). In this study, the changes in the abovementioned inflammatory markers after device closure for patients with ASDs who underwent these two different approaches were studied retrospectively.

## METHODS

The ethics committee of our university approved the present study. All surgical procedures and methods followed the Declaration of Helsinki. In addition, all the guardians of the patients were informed of the study and signed written consent forms.

In this study, we analyzed the clinical records of 53 pediatric patients with ASDs in our cardiac center between September 2018 and December 2018. The following preoperative routine examinations were completed in all patients: chest radiography, TTE, blood routine and biochemistry tests, and collection of inflammatory markers (CRP, PCT, and IL-6). All patients were diagnosed with a secundum ASD with the presence of adequate margins. Patients with other congenital heart diseases, severe pulmonary hypertension, chronic cardiac insufficiency, or inability to provide informed consent were excluded from this study.

Before choosing the different treatments for ASD closure, all the guardians of the patients were informed of the advantages, disadvantages, indications, and contraindications associated with each treatment. Considering the patient’s condition and the guardian’s willingness, the appropriate treatment was chosen for each patient. According to the different device closure procedures, the patients were divided into two groups: 19 patients who underwent transthoracic device closure were assigned to group A and 34 patients who underwent transcatheter device closure were assigned to group B.

We retrospectively collected inflammatory cytokine data from the preoperative day as well as the first, second, and third postoperative days (preOP, POD1, POD2, and POD3, respectively). In addition, patients with significantly increased inflammatory cytokine levels preoperatively and definitive diagnoses of pulmonary infection postoperatively were also excluded from this study. Postoperative pulmonary infections were defined according to the following symptoms: 1) surface temperature > 38.5ºC; 2) chest radiographs showing marked shadows in the lungs; 3) obvious moist rales detected by lung auscultations; and 4) sputum culture indicating bacterial infection. Patients with preoperative pulmonary infections must have been cured prior to further device treatment. All the above medical data were recorded in detail in the hospital database. We also analyzed the following data: operation time, incision length, and hospitalization days.

In group A, transthoracic device closure of the ASD was performed under general anesthesia. A supine position with right chest elevation was used for all patients. An incision (approximately 2~3 cm in length) was made through the 4^th^ intercostal space on the right side of the chest. After entering the thoracic cavity, the pericardium was suspended to expose the surgical field of the right atrium. Then, a purse-string suture was placed in the right atrium. The right atrium was punctured in the center of the purse-string suture, and a delivery sheath was placed. TTE was performed to track the movement of the delivery sheath from the right atrium through the ASD to the left atrium. An occluder was released along the delivery sheath. After checking via TTE that the occluder was firmly fixed without a residual shunt and that it did not affect the valve function, the delivery sheath was removed.

In group B, transcatheter device closure was performed under general anesthesia. A supine position was used in this group; then, a right femoral vein puncture was performed, and a multifunctional catheter was placed along the puncture point. TTE was used to guide the passage of the multifunctional catheter from the ASD into the left atrium. Then, a guide wire was placed along the multifunctional catheter. After the multifunctional catheter was removed, a delivery sheath was placed along the guide wire, and the inner core was removed. An occluder was placed into the delivery sheath and released to close ASD. Finally, TTE confirmed that the position of the occluder was stable without a residual shunt and without affecting valve function.

Data analysis was conducted with the Statistical Package for the Social Sciences (SPSS) software, version 13.0. Continuous data are expressed as mean±standard deviation. The independent samples *t*-test was used to compare the data between the two groups. *P*<0.05 was considered statistically significant.

## RESULTS

All patients were successfully occluded without any severe complications, such as death, low cardiac output syndrome, a cerebrovascular accident, malignant arrhythmia, multiple organ dysfunction, and the dislodgement of the occluder that would require an emergency operation. There was no significant difference in morbidity or mortality between the two groups. No vasoactive drugs were used in any of the patients.

There were no significant differences in age or weight or in the diameter of ASD in these two groups. However, the transthoracic approach was used for young patients and those with low body weight. The corresponding clinical data are shown in Table 1. Compared with the transthoracic approach, the transcatheter approach used in group B resulted in a shorter hospital stay, and there was no incision. There were similar operation times and intensive care unit stays in both groups (Table 2).

**Table 1:** Preoperative data comparison between two groups of patients.

Item	Group A	Group B	*P*-value
**N**	19	34	
**Gender (male/female)**	07/12	11/25	
**Age (years)**	8.6±4.26	10.0±5.54	0.35
**Weight (kg)**	17.0±7.1	18.5±8.0	0.50
**Pulmonary hypertension (mm Hg)**	30.0±8.2	32.3±5.3	0.22
**Size of ASD (mm)**	12.5±3.8	11.9±3.9	0.66
**Cardiothoracic ratio**	0.53±0.12	0.51±0.07	0.57

ASD=atrial septal defect

**Table 2:** Perioperative and postoperative data comparison between two groups of patients.

Item	Group A	Group B	*P*-value
Operation time (min)	46.0±6.2	43.5±8.5	0.28
Intensive care unit time (h)	7.4±2.6	7.1±2.1	0.63
Incision length (cm)	3.0±1.2	0	
Hospital stay (days)	4.9±1.3	2.4±0.8	< 0.01

The mean values of inflammatory cytokines on preOP and on POD1, POD2, and POD3 after performing the two different treatments are shown in Table 3. Compared with the preoperative levels, the postoperative levels of inflammatory cytokines were significantly increased (*P*<0.05). All the inflammatory markers reached a peak on POD1, followed by a slow decline in group A. However, in group B, except for PCT, the other three markers (CRP, WBC, and IL-6) reached a peak on POD1; then, the levels slowly decreased and basically returned to normal on POD3. There was a slight increase in PCT during the postoperative period, but there was no significant difference between the preoperative and postoperative levels. In addition, compared with the preoperative levels, the corresponding postoperative levels of inflammatory cytokines in the transthoracic group were significantly higher than those in the transcatheter group (*P*<0.05).

**Table 3:** Changes in the levels of inflammatory markers of patients in groups A and B.

Item		preOP	POD1	POD2	POD3
WBCs	A	7.9±1.1	14.5±3.7	13.4±1.9	11.7±1.6
	B	7.4±2.8	11.2±4.0	9.4±2.7	8.1±1.8
	P	0.51	< 0.01	< 0.01	< 0.01
PCT	A	0.17±0.14	3.8±1.72	2.1±1.07	1.1±0.86
	B	0.15±0.11	0.28±0.16	0.24±0.13	0.22±0.10
	P	0.18	< 0.01	< 0.01	< 0.01
CRP	A	0.78±0.37	33.68±22.8	24±16.5	12.74±6.2
	B	0.88±0.41	10.8±5.05	6.02±1.43	1.1±3.9
	P	0.39	< 0.01	< 0.01	< 0.01
IL-6	A	1.88±1.14	57.2±23.1	31.1±17.4	23.7±12.3
	B	1.56±0.81	15.4±10.6	10.8±9.7	2.6±1.7
	P	0.25	< 0.01	< 0.01	< 0.01

CRP=C-reactive protein; IL-6=interleukin-6; PCT=procalcitonin; POD1=first postoperative day; POD2=second postoperative day; POD3=third postoperative day; preOP=preoperative day; WBCs=white blood cells A=group A; B=group B; P=comparison between these two groups in the same period

## DISCUSSION

The traditional surgical repair of ASDs has been widely used and effective, but it still requires the use of CPB, with a large incision and slow postoperative recovery^[2-4]^. Transthoracic and transcatheter device closures of ASDs have been increasingly reported in recent years; the previous reports also confirmed that there were low morbidity and mortality in both treatments^[5,6]^. With the accumulation of experience and the progression of technology, these two treatments have tended to replace surgical repair for the treatment of ASDs^[10]^. Transcatheter device closure of ASDs has the advantages of no incision and a short hospitalization time^[11,12]^. However, in the treatment of ASDs with the transcatheter approach, due to the limitation of the femoral vein and long interventional path, age and peripheral vascular condition are strict requirements^[13]^. Transthoracic minimally invasive device closure of ASDs is another treatment that combines the advantages of surgical and interventional techniques. This approach uses a short operative path, has excellent controllability, is easy to learn and operate, and is not limited by age or weight. Although the longer hospital stays might lead to higher medical costs, the magnitude of the increase was not statistically significant. In our cardiac center, we routinely use these two device methods for secundum ASD closure.

Murat Guvener et al.^[14]^ reported that the systemic inflammatory response was associated with body surface area, body weight, CPB time, and aortic occlusion time after surgery for congenital heart disease. Polomsky M et al.^[15]^ demonstrated that stimulation of the heart during surgery could cause systemic inflammatory response syndrome (SIRS) and the corresponding clinical symptoms. Therefore, we can conclude that acute systemic inflammation after cardiac surgery is the result of many related markers, such as blood exposure to nonphysiological surfaces, surgical trauma, and myocardial ischemia-reperfusion^[16,17]^. In this study, CPB and aortic clamps were not required for either of the two procedures. According to the literature, there have been few studies on whether transthoracic and transcatheter device closure of ASDs can cause a systemic inflammatory reaction. Therefore, we designed this study to focus on the changes in inflammatory markers in children who underwent device closure of ASDs with these two approaches.

Cardiac surgery is still associated with a specific incidence of postoperative nosocomial infection. According to some studies, the infection rate is 5.0%~21.0%^[18]^. If postoperative infection cannot be controlled in time, once it develops into SIRS, the mortality rate increases significantly^[14]^. Therefore, rational use of antibiotics as early as possible after surgery and appropriate changes in medicines according to inflammatory indicators can effectively prevent the possibility of postoperative infection. The inflammatory cytokines commonly used to treat infection are WBCs, CRP, PCT, and IL-6. WBC counts are often used to diagnose bacterial infections. Although the medical normal range of WBCs is extensive and differences in the WBC counts among individuals are evident, WBC count, as the most commonly used and classic inflammatory indicator in clinical practice, was still used in this study. CRP is a typical acute time-phase protein in acute inflammation, and CRP is elevated during tissue damage, immune responses, and inflammation; CRP also occurs early and fast and changes with the severity of systemic inflammation^[19,20]^. PCT is a precursor of calcitonin, belongs to the glycoprotein family, and has no hormonal activity. Studies have shown that lipopolysaccharides are the main pathogenic component stimulating the elevation of PCT, which makes PCT highly specific^[21,22]^. Li et al.^[22]^ reported that PCT was more accurate than WBC count and CRP for predicting early postoperative infection after pediatric cardiac surgery. Therefore, PCT has unique advantages in distinguishing infectious and noninfectious SIRS^[23]^. IL-6 is an essential mediator of inflammation and is generated by many cells. In addition, Steinberg JB suggested that the production of IL-6 was associated with surgery and that there was a similar IL-6 elevation after heart surgery, regardless of whether CPB was performed^[24]^. Numerous studies have shown that IL-6 plays a role in acute inflammation^[23-25]^. Therefore, we chose WBCs, CRP, PCT, and IL-6 as our research indicators in this study.

According to the laboratory results of this study, there was a significant systemic inflammatory reaction after transthoracic device closure of ASDs, and this reaction was most evident on POD1. A systemic inflammatory reaction was also observed after transcatheter device closure of ASDs. However, the intensity of the response was significantly less evident with the transcatheter approach than with the transthoracic approach, and there was no significant change in PCT compared with the preoperative data. Du Bin et al.^[23]^ reported that PCT was more reliable than other inflammatory indicators such as CRP and WBC in terms of differentiating infection from noninfection. Therefore, our results also suggest that PCT may be an indicator for distinguishing the systemic inflammatory response caused by disease and noninfectious markers after the transcatheter approach; however, further studies are needed to confirm this result. In addition, on the POD3 of transcatheter procedure, inflammatory cytokines decreased, returning to a normal range. However, the level of inflammatory cytokines on the POD3 of transthoracic approach was still higher than the preoperative levels, which suggests that the inflammatory response associated with the transthoracic approach lasts longer than the response associated with transcatheter approach. In addition, this study also illustrated that the levels of inflammatory cytokines were still significantly higher than the average levels on the POD3 of transcatheter device procedure; thus, the possibility of postoperative infection should be considered.

The differences in age and weight of the children in group B were more significant than those in group A, although these differences were not statistically significant, which may be one of the reasons that the systemic inflammatory response after transcatheter approach was weaker than that after transthoracic approach. Further research with more patients is needed to confirm these results. Surgical incisions in transthoracic approach may also lead to inflammation. Additionally, the author inferred that the pericardium should be opened using a transthoracic approach and that puncture directly from the surface of the heart may directly cause damage to the atrium^[26]^, which may be another reason to the fact that the inflammatory reaction after transthoracic approach was stronger than that after transcatheter approach.

This study was limited by its retrospective nature, but it still has some clinical implications. This is a single-center observational study with a limited number of patients, and only four inflammatory markers were selected as postoperative inflammatory indicators. The grouping of the patients was not random; although the two groups of patients were homogeneous, there was still selective deviation. Further prospective, randomized, double-blind controlled trials should be completed to confirm this conclusion. In addition, the reason for the difference in postoperative inflammatory responses between these two device treatments requires further study.

## CONCLUSION

Both transthoracic and transcatheter device closures of ASDs produce a systemic inflammatory reaction. However, the intensity of the systemic inflammatory response after transthoracic approach is stronger than that after transcatheter approach, and a combination of other clinical manifestations are required to distinguish infection from noninfection.

**Table:** 

Authors' roles & responsibilities	
ZNH	The acquisition, analysis, or interpretation of data for the work; final approval of the version to be published
JSH	The acquisition, analysis, or interpretation of data for the work; final approval of the version to be published
KPS	The acquisition, analysis, or interpretation of data for the work; final approval of the version to be published
ZRL	The acquisition, analysis, or interpretation of data for the work; final approval of the version to be published
QC	Substantial contributions to the conception or design of the work; or the acquisition, analysis, or interpretation of data for the work; final approval of the version to be published
